# The Neutrophil-to-Lymphocyte Ratio as a Biomarker in Cutaneous Oncology: A Systematic Review of Evidence beyond Malignant Melanoma

**DOI:** 10.3390/cancers16051044

**Published:** 2024-03-04

**Authors:** Konstantinos Seretis, Konstantinos Sfaelos, Elena Boptsi, Georgios Gaitanis, Ioannis D. Bassukas

**Affiliations:** 1 Department of Plastic Surgery, Faculty of Medicine, School of Health Sciences, University of Ioannina, 451 10 Ioannina, Greece; drseretis@uoi.gr (K.S.); elenabop53@gmail.com (E.B.); 2 Department of Skin and Venereal Diseases, Faculty of Medicine, School of Health Sciences, University of Ioannina, 451 10 Ioannina, Greece; k.sfaelos@uoi.gr (K.S.); ggaitan@uoi.gr (G.G.)

**Keywords:** hematological biomarkers, skin cancer, non-melanoma skin cancer, keratinocyte skin cancer, cutaneous lymphoma, cutaneous sarcoma, neutrophil-to-lymphocyte ratio

## Abstract

**Simple Summary:**

This systematic review concludes that a higher neutrophil-to-lymphocyte ratio (NLR), a robust indicator of adverse prognosis across the common metastatic cancers including malignant melanoma, is also a promising biomarker of tumor aggressiveness in all advanced stage non-melanoma skin cancers. A distinct exception with still inconclusive evidence is the cutaneous T-cell lymphoma, although there are some data indicating that higher NLR may predict poorer prognosis in patients with earlier stages mycosis fungoides. Finally, given the promising data concerning the impact of NLR as a prognosticator of the outcome of the treatment with the current immunotherapies, there is presently a need for studies focusing on the role of NLR in the framework of the treatment of advanced non-melanoma skin cancer.

**Abstract:**

With the ongoing progress of basic research along with the introduction of new pharmaceutical options spanning almost all therapeutic areas, the need for biomarkers that will be implemented into the personalized medical approach is higher than ever. Their use can be incorporated into clinical practice and can be applied to the classification of disorders and the evaluation of disease severity but also to the monitoring of the progress of therapeutic/pharmaceutical interventions. This systematic review collects the findings of hematologic biomarkers in various cutaneous malignancies, excluding malignant melanoma, to support their potential use in the prognosis but also in the assessment of therapeutic strategies for the specific category of skin disorders.

## 1. Introduction

Several studies have highlighted the biomarker utility of some remarkably simple hematological indexes that can be derived from routines in clinical practice complete blood counts. Particularly, the neutrophil-to-lymphocyte ratio (NLR), calculated as the quotient of the absolute numbers of neutrophils to lymphocytes, has been evaluated as a predictor of disease course and outcome in a variety of medical conditions, including many cancers [1]. In healthy individuals, average NLR estimations range between 1.7 and 2.0, with most people presenting with values from 1.0 to 3.0 [2,3,4]. Notably, within this estimated normal range, NLR values tend to increase with age and they are also slightly, but significantly, higher in males compared to females (mean 1.88 vs. 1.68, respectively) [4]. Central to understanding the pathophysiology of NLR are findings showing that higher index values are independently linked to significantly increased all-cause mortality, as well as to the disease-specific mortality of major medical conditions, including heart, chronic lower respiratory, and kidney diseases, in the general population [4,5]. Generally, from a mechanistic point of view, both an increase in neutrophil or a decrease in lymphocyte counts may result in an NLR increase; however, two scenarios prevail in clinical practice: (a) an isolated rise in neutrophil counts, or, more frequently, (b) an increase in neutrophils parallel to a lymphocyte decline [6]. Within this latter scenario, an increased NLR is interpreted as a proxy for the burden of systemic inflammation in the body, a state of homeostasis disruption which is associated with increasing frailty (nutritional, functional, and immunological decline) and characteristic laboratory aberrations in circulating white blood cells, notably concomitant neutrophilia and relative lymphocytopenia (translating into an NLR increase) [7]. In other words, it is suggested that NLR, as an index, integrates the impact of the two crucial aspects of the immune system: the inherent (innate) and adaptive immune responses, mainly represented by the functions of neutrophils and the lymphocytes, respectively, thus reflecting the body’s underlying condition that determines their mutual homeostasis [5].

Since its evaluation as a promising prognostic factor in colorectal cancer almost 20 years ago [8], NLR has been established as an independent prognosticator for various types of cancer regardless of therapy, as well as in different settings of clinical oncology, including response to treatment. As a rule, a higher NLR is associated with poorer survival and a lower probability of response to immunotherapy, both at a pan-cancer level and within several cancer types [9,10,11]. However, NLR was associated with cancer mortality only in the first follow-up year (HR 1.48, 95%CI 1.11–1.98), implying also that NLR is more consistently predictive of increased mortality only in the more advanced and aggressive cancer cases, such as in those patients who require chemotherapy or who have an inoperable disease [5]. Moreover, as intuitively anticipated, NLR may be severely biased and therefore not a meaningful biomarker in certain cases, particularly in hematologic malignancies [12]. As in most serious diseases, so also in the setting of malignant diseases, an NLR increase usually represents concurrent absolute neutrophilia and lymphopenia in the framework of the “cancer-associated inflammation”; the result of a complex pathophysiologic interaction between the tumor and its host and a key determinant of disease progression and survival in most cancers [13]. Neutrophilia, with elevated absolute numbers of circulating neutrophils, is a common finding in cancers, and has been associated with poor disease outcomes, such as shorter survival [14,15]. Moreover, myeloid-derived suppressor cells, the NLRP3 inflammasome, neutrophil extracellular traps, and absolute neutrophil count tend to be directly related to the NLR [16,17,18]. Interestingly, a meta-analysis of 25 cancer types identified neutrophils as the immune cell type with the strongest correlation to poor disease outcomes [19] and reviews of pertinent clinical data make a strong empirical argument for an important growth promotion by neutrophils in the most prevalent cancer types [20,21]. Neutrophilia seems to be a core, but not the only, laboratory feature that underlies increased NLR values. The number and functional integrity of lymphocytes is also inevitable for effective tumor growth control. Intact lymphocyte function is necessary for tumor surveillance and destruction, and neutrophils may also implement their pro-neoplastic role indirectly by suppressing lymphocyte proliferation and inducing lymphocyte apoptosis, thus contributing to the characteristic lymphopenia of cancer-associated inflammation [22]. Moreover, an increased NLR may also reflect the adaptive response of the patient’s body to a growing tumor, including protection against tumor-induced cachexia [23]. 

The majority of studies that have evaluated the prognostic role of NLR in cutaneous malignancies have focused on malignant melanoma, with results clearly highlighting a strong association between an elevated NLR and worse prognosis (poor overall and disease-free survival) in patients with localized, high-risk, and metastatic disease. Moreover, a baseline NLR is a promising prognostic biomarker for treatment response in melanoma patients receiving immunotherapy, and also, an agnostic, modality-independent predictive factor of shortened overall survival and progression-free survival in metastatic melanoma, treated with either metastasectomy or immunotherapies. Review papers and meta-analysis studies have compiled the pertinent data on this growing body of evidence [22,24,25,26,27,28,29,30,31,32] and will not be reviewed again herein. Herewith, to supplement the evidence of the use of the NLR (and allied hematological biomarkers) in cutaneous oncology, we aim to systematically review, and consequently summarize the evidence on, their application in assessing prognosis, survival, and treatment outcomes in patients with skin malignancies other than malignant melanoma.

## 2. Materials and Methods

A predetermined protocol was established according to the Cochrane Handbook’s recommendations [33] and registered at the PROSPERO database (registration number: CRD42024500121). The systematic review adhered to the updated PRISMA (preferred reporting items for systematic reviews and meta-analyses) guidelines, presented in the Supplementary Materials (Table S1) [34]. 

### 2.1. Search Strategy

An electronic literature search in MEDLINE (PubMed), Scopus, the Cochrane Library, and the CENTRAL electronic databases was conducted from inception to 31 December 2023. The string search “neutrophil-to-lymphocyte” and “skin cancer” was applied and restricted to the fields ‘Title’ and ‘Abstract’. No time and language restrictions were applied. The complete search strategy is included in the Supplementary Materials (Table S2). The citation lists of retrieved articles were screened manually to ensure the sensitivity of the search strategy and to identify additional relevant material. 

### 2.2. Eligibility of Relevant Studies 

Studies met the following inclusion criteria: (1) report of the neutrophil-to-lymphocyte ratio; (2) reported data on certain skin cancer entities, i.e., keratinocyte skin cancers, including epithelial precancer and carcinoma in situ, other non-melanoma skin cancers (NMSC), cutaneous sarcomas, and skin lymphomas; and (3) publication in a peer-reviewed journal. We excluded studies reporting on melanoma; studies not reporting NLR values in NMSC patients; and review articles, duplicate reports, editorials, and non-human studies were also excluded. 

### 2.3. Study Selection 

Two reviewers (K.S. and E.B.), independently performed the literature search and screened retrieved database files and the full texts of potentially eligible studies for relevance. Disagreement was resolved by consensus.

### 2.4. Data Collection and Risk of Bias Assessment 

Data extraction was conducted independently by the 2 reviewers using a standardized form. Discrepancies were resolved by consensus. We extracted data, including the general study characteristics, patient demographics, and outcomes of interest. Primary outcome was the values of the peripheral blood NLR, which will enable its application as prognostic or diagnostic biomarkers for cutaneous neoplasms. 

The quality of the studies was assessed using the MINORS tool for non-comparative studies (Table S3) [35] and the Cochrane risk of bias tool (ROBINS-I, Table S4) for non-randomized comparative studies [36]. The quality assessment of the included studies is available in the Supplementary Materials (Methods, Tables S4 and S5). 

### 2.5. Data Synthesis and Analysis

We provided a narrative summary of the included studies based on the cutaneous neoplasm type and the publication date. Data were consolidated and subsequently summarized with descriptive statistics.

## 3. Results

**Figure 1:**
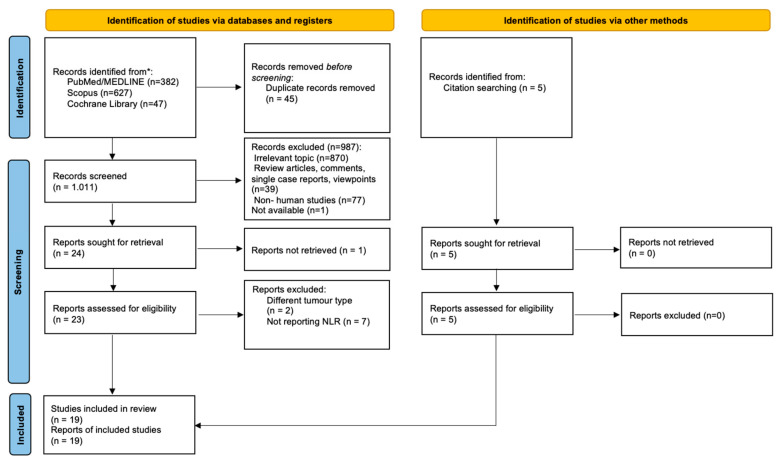
PRISMA flowchart for study selection. * For search details see Section 2.1.

The study selection process is summarized in Figure 1. From a total of 1061 records, 19 studies were eligible and included in the data analysis. 

The 19 studies included were conducted in Japan (6), Italy (4), Turkey (3), the USA (2), Germany (2), France (1), and New Zealand (1). All studies were published after 2016 and, apart from one prospectively designed study, the rest were retrospective. This review identified the neutrophil-to-lymphocyte ratio as the hematological biomarker most widely employed in the dermatological oncology. The NLR was utilized for cutaneous squamous cell carcinoma (cSCC, 4 studies), extramammary Paget disease (3 studies), Merkel cell carcinoma (4 studies), cutaneous angiosarcoma (2 studies), and mycosis fungoides/primary cutaneous T cell lymphoma (5 studies). In addition, one study compared biomarker levels at tumor diagnosis between patients with basal cell carcinoma (BCC), cSCC and cutaneous malignant melanoma (cMM). Table 1 compiles the pertinent information of the 19 reviewed studies.

The results are presented according to the type of skin cancer.

### 3.1. Cancer of Epithelial Cell Origin

#### 3.1.1. Keratinocyte Skin Cancer

Keratinocyte skin cancer (KSC, largely the same disease family with the former category of non-melanoma skin cancers) is the umbrella term used to designate a clinicopathological spectrum of malignancies that all originate from the keratinocytes of the epidermis and the epithelium of skin adnexals. KSC is a heterogeneous tumor family that includes basal cell carcinoma (BCC), the most frequent entity within KSC, as well as the different variants of the cutaneous squamous cell carcinomas (cSCC), including SCC in situ (or Bowen’ disease). KSCs are by far the most prevalent malignant diseases and a major global health challenge as they have recently been estimated to be responsible for as many disease-specific deaths worldwide as malignant melanomas [37]. The mainstay of their treatment is the radical surgical excision, which has translated to notably high cure rates. This is particularly true for the common biologically quite indolent BCC, which is only in exceptional cases aggressive. Accordingly, the prognosis for KSC is comparatively favorable; a fact that may partly explain the rarity of published studies that address the use of hematological biomarkers to predict patients’ clinical course. This is the case, also compared to the much rarer, though with a generally worse prognosis, cutaneous malignant melanoma.

##### Basal Cell Carcinoma (BCC)

One relevant study was found. Derebaşınlıoğlu et al. [38] compared the NLR, the platelet-to-lymphocyte ratio (PLR), the monocyte-to-lymphocyte ratio (MLR), and the systemic inflammation index (SII = platelets count × NLR) of patients with the three most frequent skin cancer types (BCC, cSCC, and cMM). The systemic inflammation burden at diagnosis according to the studied biomarkers was lowest in patients with BCC and highest in those with cSCC. Indicatively, the mean ± standard deviation of the NLR of BCC patients (*n* = 144) was, at 2.53 ± 1.53, significantly lower than that of patients with cSSC (*n* = 84, 4.41 ± 5.25; *p* = 0.001). The NLR values of patients with cMM (*n* = 29) were intermediate (3.25 ± 3.19).

##### Cutaneous Squamous Cell Carcinoma (cSCC) 

Cutaneous squamous cell carcinoma (cSCC) is the second most common KSC after BCC, accounting for 20% of cutaneous malignancies and about 75% of all deaths due to skin cancer, excluding cutaneous malignant melanoma [39].

In a retrospective study of 168 patients diagnosed with cSCC, of whom 129 with and 39 without therapeutic immunosuppression, Seddon et al. [40] found that the basal median NLR was statistically significantly higher in immunosuppressed (median: 5.4, range: 1.1–46.0) compared to immunocompetent patients (median: 3.0, range: 0.7–23.0; *p* < 0.001). Among the 39 patients without therapeutic immunosuppression, four developed metastases during follow-up. Notably, all of them had a baseline NLR ≥ 3.0 [40]. A single-center retrospective case series study from Japan investigated the association between NLR values, disease-specific survival, and sentinel lymph node (SLN) positivity in patients with cSCC. Of the 222 included patients, 147 had surgical treatment, and 50 of these additionally had an SLN investigation. Using univariate and multivariate analyses, the authors identified an elevated NLR as an independent prognostic factor for poor disease-specific survival and as a predictive factor for SLN positivity. Despite the limitation of the small number of participants, they concluded that NLR is a useful biomarker to predict prognosis in cSCC [41]. In a cohort study including 30 frail, elderly, or immunosuppressed patients with locally advanced (*n* = 25) or metastatic cSCC (*n* = 5), Stripoli et al. [42] observed that a low baseline NLR and PLR (platelet-to-lymphocyte ratio), correlated with a better response to cemiplimab treatment. Moreover, in patients under treatment, a progressive NLR increase distinguished non-responders from treatment responders [42].

In a retrospective study with 51 newly diagnosed cSCC patients, Di Raimondo et al. [43] evaluated the correlation of NLR with the stage of the disease. With a median NLR = 2.2, the NLR values were significantly lower in non-advanced disease stages (in situ and stage I), compared to more advanced (stages > I) of cSCC (NLR = 4.87). Moreover, they reported that an NLR > 3.07 highly predicted an advanced stage of cSCC.

#### 3.1.2. Cutaneous Adenocarcinoma

Extramammary Paget’s disease (EMPD) is a rare, usually primary, adenocarcinoma of the skin that originates from the epithelium of sebaceous gland ducts or even more primitive ancestor keratinocyte stem cells. In most cases, EMPD begins as an intraepithelial adenocarcinoma and may develop to a therapeutic challenging tumor with variable disease aggressiveness [44,45]. The predictive role of the NLR was addressed in a recent, retrospective, single-center case series of 109 patients with EMPD (*n* = 45 of them with metastatic disease) from Japan. Significantly higher NLR values were found in patients with metastatic compared to non-metastatic EMPD at the time of evaluation (mean: 3.39 vs. 2.53; *p* < 0.005). Moreover, the NLR was an independent prognostic factor for overall survival (*p* = 0.019) as well as an independent predictor of survival among EMPD patients with metastatic disease (*p* = 0.036) [46]. In a follow-up multivariate analysis that included 85 patients with EMPD, the same authors found that NLR and a higher invasion level were the two independent predictive factors for SLN biopsy positivity [47]. Similarly, Ebata et al. [48], in a retrospective study of 137 patients who underwent sentinel lymph node biopsy for EMPD, showed that an NLR > 3.0 was a predictor of lymph node metastasis (OR = 3.31, 95%CI: 1.117–9.804; *p* = 0.038).

Overall, the evaluation of the routinely, easily assessed NLR seems to be a useful, highly feasible biomarker for predicting prognosis in patients with EMPD.

### 3.2. Merkel Cell Carcinoma

The Merkel cell carcinoma (MCC) is a rare, aggressive skin cancer composed of malignant cells of neuroendocrine and epithelial differentiation. It typically presents as a rapidly growing, red–violet painless nodule on sun-exposed areas of the head and neck or at the upper extremities of elderly patients, and is associated with a 5-year survival of approximately 30–64% [49,50]. In a retrospective study of 75 patients with MCC, Zaragoza et al. [51] showed that a high NLR at baseline (≥4.0) was associated with increased disease-specific mortality in univariate (HR = 2.76, *p* = 0.023) and in multivariate (HR = 3.30, *p* = 0.020) analysis. However, NLR was not associated with the recurrence risk. Similarly, a recent study, which was presented as conference abstract, included 95 MCC patients, and showed that baseline NLR was strongly associated with a worse prognosis, i.e., decreased disease-free survival (DFS; multivariate analysis: HR = 1.63; 95%CI: 1.34–1.99, *p* < 0.001) and worse overall survival (OS; multivariate analysis: HR = 1.44; 95%CI: 1.10–1.90, *p* = 0.008) [52]. From a treatment perspective, in a prospective, multicenter phase II trial of 50 patients with advanced unresectable MCC, Nghiem et al. [53] showed that a low NLR across the first three months of therapy (but not any isolated value either at baseline or at any individual time point during therapy) was associated with improved objective response (complete or partial response; mixed, fixed-effect multivariate model, *p* = 0.043) to first-line pembrolizumab treatment, as well as with longer patients’ OS at 30 months follow-up (*p* = 0.028). A more complex index, named pan-immune-inflammation value (PIV), which combines four complete blood count-based parameters, neutrophil, platelet, monocyte, and lymphocyte counts, has been evaluated by Gambichler et al. [54] and was found to be significantly increased in MCC patients compared to a sex- and age-matched healthy control group, revealing its promising prognostic ability.

### 3.3. Cutaneous Sarcomas

Cutaneous sarcomas are the subset of the soft tissue sarcomas (STSs) that originate from the connective tissue skeleton of the skin, the dermis, and the subcutis [55,56,57]. They comprise a clinically and morphologically highly heterogeneous family of mesenchymal spindle cell tumors of varying pathogenesis, that includes clinical entities as variables in their clinical behavior as dermatofibrosarcoma protuberans, atypical fibroxanthoma, cutaneous undifferentiated pleomorphic sarcoma, leiomyosarcoma, liposarcoma, Kaposi’s sarcoma, and malignant peripheral nerve sheath tumor. Cutaneous sarcomas are rare tumors that often pose a difficult diagnostic dilemma and require a multi-disciplinary approach for appropriate management. Histology remains mandatory for correct diagnosis. Surgical resection with sufficient margins remains the gold standard treatment although ongoing dissection of their pathogenesis at molecular level promises the development of targeted therapies. Unlike the overall poor prognosis of most non-cutaneous STSs, with adequate treatment the prognosis in terms of disease-specific mortality of most dermal sarcomas is excellent with the main therapeutic challenge being their trend to exhibit local recurrences. However, biologically aggressive entities do also exist, with the most characteristic being the angiosarcomas [55,56,57]. Apart from cutaneous angiosarcomas, no studies were found in the literature that report on the association of NLR or similar hematologic biomarkers and the clinical features of cutaneous sarcomas. 

Angiosarcomas are rare soft-tissue sarcomas of endothelial cell origin that have a poor prognosis. They can arise anywhere in the body, most commonly presenting as cutaneous disease in elderly white men, involving the head and neck and particularly the scalp [58]. Two small retrospective studies from Japan have reported on the prognostic role of hematological biomarkers in primary cutaneous angiosarcomas. In a retrospective study of 26 patients with primary cutaneous angiosarcoma, a high NLR (>2.4) was an independent prognostic factor for shorter overall survival (HR = 5.04, 95%CI: 1.26–20.1; *p* = 0.022) [59]. Another, case–control study reported a statistically significant difference in NLR and PLR values between 17 patients with angiosarcoma of the face and scalp (ASFS) and 56 healthy matched controls. However, the PLR seemed to be superior to the NLR as biomarker for patients with ASFS and was proposed as a clinically useful marker in patients with small ASFS [60]. These results indicate that the NLR, and probably also the PLR, may serve as prognostic predictors in primary cutaneous angiosarcoma, probably also in other types of soft-tissue sarcoma of the skin.

### 3.4. Primary Cutaneous Lymphomas

Only studies reporting on T cell lymphomas were identified. This literature search did not return any reports linking the NLR or other hematological biomarkers with the clinical course parameters of patients with primary cutaneous B cell lymphomas. Primary cutaneous T cell lymphoma (CTCL) is a chronic, relapsing illness, categorized as a rare type of extranodal non-Hodgkin’s lymphoma that primarily affects the skin. The uncertain pathogenesis and variable clinical presentation make the diagnosis and management of CTCL a challenge. Mycosis fungoides (MF) comprises more than half of all CTCLs and is a generally indolent disease with a low tumor burden in the early stages. Treatment focuses mainly on symptomatic relief and improving the patient’s disease-related quality of life [61]. However, in a subset of patients, the disease advances rapidly, with relatively early malignant T cell infiltration of extracutaneous tissues and an overall worse prognosis. To date, beyond the actual disease’s skin stage, there are still no reliable biomarkers for the timely identification of patients at a higher risk of early disease spread [62].

A study compiling 117 patients with MF did not find any relationship between their NLR values and indication for systemic treatment (PUVA and/or chemotherapy), stage progression, or time to stage progression [63]. Similarly, a baseline NLR did not appear to serve as suitable biomarker for the prediction of treatment outcome with extracorporeal photopheresis and patient survival in another retrospective study that included 29 MF patients [64]. Also, a study of a cohort of 115 patients (98 MF, 17 other CTCL) showed that the NLR was not significantly associated with prognosis. Remarkably, a greater than normal copper serum level was present in nearly 20% of patients and was associated with an increased risk of disease progression and the shortened disease-specific survival of patients with patch or plaque stage MF, but not of those with tumor stage MF or erythrodermic CTCL [65].

Conversely, a retrospective study that reviewed 119 MF patients concluded that the mean NLR was higher in the patients compared to healthy controls (mean ± SD: 2.07 ± 1.17 vs. 1.76 ± 0.53; *p* < 0.05). Moreover, NLR values > 2.85 at diagnosis were positively correlated with advanced disease stage and disease progression, indicating that a high NLR at diagnosis is a poor prognostic factor for identifying high-risk patients with MF [66]. Similarly, in a recent study from Italy, Di Raimondo et al. [67] analyzed the association of baseline NLR before confirming MF diagnosis and the clinical disease characteristics of 302 patients presenting with variable disease stages. Applying the cut-off value of NLR = 2.3 (calculated using the ROC procedure), they found that a higher NLR is associated with allocation to a high-grade disease stage (stages higher than IIB, *p* = 0.001); however, not with the risk of stage progression (*p* = 0.077).

**Table 1:** Compilation of studies reporting on the association of NLR with the clinical features of skin cancers, other than malignant melanoma.

Skin Cancer	Study			Entity/Patients (*n*)		Results
	Author/Year *	Country	Type		End Point	Outcome
KSC	Di Raimondo/2022 [43]	Italy	RS	cSCC/51	Tumor stage	NLR > 3.07 at diagnosis was associated with a higher than stage I
	Strippoli/2021 [42]	Italy	RS	advanced-metastatic cSCC/30	Response to cemiplimab	Low baseline NLR correlated with a better treatment response
	Seddon/2016 [40]	New Zealand	RS	cSCC/168	Patient’s immune state	NLR was higher in immunosuppressed compared to immunocompetent patients (NLR = 3.0, range: 0.7–23.0; *p* < 0.001)
	Derebaşınlıoğlu/2022 [38]	Turkey	RS	BCC/144 cSCC/84	Differential diagnosis	NLR was higher in cSCC compared to BCC patients
					Tumor location	No statistical differences in NLR according to location
					LN metastases	Higher PLR levels (>180.7) and PltxNLR (>747) were correlated with higher risk of LN metastasis at the time of initial diagnosis
	Maeda/2022 [41]	Japan	RS	cSCC/222	Disease-specific survival	An elevated NLR was an independent prognostic factor for adverse DSM
					SLN metastasis	An elevated NLR was a predictive factor for SLN positivity
Cutaneous adeno-carcinoma	Maeda/2022 [46]	Japan	RS	EMPD/109	Metastases	Higher NLR in patients with metastatic EMPD (mean: 3.39 vs. 2.53; *p* < 0.005)
					OS	NLR was an independent prognostic factors for OS (*p* = 0.019)
	Maeda/2023 [47]	Japan	RS	EMPD/85	SLN metastasis	NLR was independent predictor of SLN positivity
	Ebata/2021 [48]	Japan	RS	EMPD/137	LN metastases	NLR > 3.0 was a predictor of LN metastasis (OR = 3.311, 95%CI: 1.117–9.804; *p* = 0.0380)
MCC	Zaragoza/2016 [51]	France	RS	MCC/75	Disease specific mortality	Baseline NLR ≥ 4 is independently associated with DSM (multivariate analysis; HR = 3.30, 95%CI: 1.21–9.01, *p* = 0.020)
					Recurrence	NSA
	Torchio/2020 [52]	Italy	RS	MCC/95	DFS	Baseline NLR was strongly associated with worse DFS (multivariate analysis: HR = 1.63, 95%CI: 1.34–1.99; *p* < 0.001)
					OS	Baseline NLR was strongly associated with worse OS (multivariate analysis: HR = 1.44, 95%CI: 1.10–1.90; *p* = 0.008)
	Nghiem/2021 [53]	USA	PS	Advanced unresectable MCC treated with pembruli-zumab/50	Treatment response	NLR across the first 3 months of therapy (but not any isolated value) correlated with improved objective response (*p* = 0.043)
					OS	NLR across the first 3 months of therapy (but not any isolated value) correlated with 30 months OS (*p* = 0.028)
	Gambichler/2022 [54]	Germany	RS	Stage I-III MCC/49	Tumor stage	PIV was higher in MCC stage II and III in comparison to MCC stage I
					Recurrence	PIV >372 was significantly associated with MCC recurrence (*p* < 0.0001)
Primary cutaneous sarcomas	Awaji/2021 [59]	Japan	RS	PCA/26	OS	NLR > 2.4 was an independent prognostic factor for shorter OS (hazard ratio = 5.04, 95%CI: 1.26–20.1; *p* = 0.022)
	Suzuki/2017 [60]	Japan	RS	ASFS/17	Diagnosis	Higher NLR in patients (2.63 ± 0.94 in patient vs. 1.93 ± 0.81 in the control group; *p* = 0.0063)
				Healthy controls/56		
Primary cutaneous lymphomas	Eren/2016 [63]	Turkey	RS	MF/117	Treatment indication	NSA
					Progression in stage	NSA
					Time to progression	NSA
	Gambichler/2022 [64]	Germany	RS	MF/29	Treatment outcome	NSA
					Patients’ survival	NSA
	Vonderheid/2019 [65]	USA	RS	MF/98 Other/17	Prognosis	NSA
	Cengiz/2017 [66]	Turkey	RS	MF/119	Diagnosis	Higher NLR in MF patients compared to controls (2.07 ± 1.17 vs. 1.76 ± 0.53; *p* < 0.05)
					Disease stage and progression in stage	NLR > 2.85 at diagnosis positively correlates with advanced disease stage and progression in stage
	Di Raimondo/2023 [67]	Italy	RS	MF/302	Disease stage and progression in stage	NLR > 2.3 is associated with higher disease stage (>IIB), however, not with the risk of patients’ progression in stage (*p* = 0.077).

* The numbers refer to the list of references. Abbreviations: 95%CI: 95% confidence interval; KSC: keratinocyte skin cancer; RS: retrospective study; PS: prospective study; ASFS: angiosarcoma of the face and scalp; CR: complete response; BCC: basal cell carcinoma; cSCC: cutaneous squamous cell carcinoma; CTCL: cutaneous T cell lymphoma; DFS: disease-free survival; DSM: disease-specific mortality; EMPD: extra-mammary Paget disease; HR: hazard ratio; LN: lymph node; MCC: Merkel cell carcinoma; PLR: platelet-to-lymphocyte ratio; MF: mycosis fungoides; NLR: neutrophil-to-lymphocyte ratio; NSA: no significant association; OR: odds ratio; PIV: Pan-immune-inflammation value; OS: overall survival; PCA: primary cutaneous angiosarcoma; PR: partial response; SLN: sentinel lymph node.

### 3.5. Evaluation of Different Biomarkers

Core results per tumor type and hematological biomarker are summarized in Table 2. Data on the NLR are reported for all studied tumor entities. In addition, PLR data that are in line with the corresponding NLR data are presented for cSCC and for cutaneous angiosarcomas. Moreover, one study reported exclusively on the application of a more complex hematological biomarker, the PIV index, in patients with MCC.

## 4. Discussion

Cutaneous oncology remains at the forefront of research interest, due to the globally exceptionally high prevalence of the keratinocyte skin cancers and, also, the distinct aggressiveness of certain other types, like MCC and angiosarcomas. Despite being in most cases easily visible, the often asymptomatic course renders timely diagnosis in many instances difficult. However, early detection and intervention remain the mainstay of the current management of skin cancer, crucial to minimize potential tumor consequences, which emphasizes the importance of effective diagnostic and prognostic biomarkers. Prognostic biomarkers can delineate the profile of tumors, providing insights into the dynamics of cancer progression and the potential of metastatic expansion, helping clinicians tailor therapeutic interventions in a personalized manner. Diagnostic biomarkers, on the other hand, play a crucial role in early detection of primaries and of relapses, allowing for timely and targeted implementation of therapeutic measures. Research efforts are focused on identifying biomarkers that can be reliably assessed through non-invasive methods. The evidence from colorectal cancer and melanoma highlights the strong association of elevated NLR values with worse prognosis in patients with localized, high-risk, and metastatic disease [8]. Similarly, a baseline NLR has a promising prognostic value for the treatment response in melanoma patients receiving immunotherapy, and for the approach to the risk of shortened overall survival and progression-free survival in metastatic melanoma, treated with either metastasectomy or immunotherapies [24,25,26,27,28,29,30,31,32].

This systematic review focused on cutaneous tumors other than melanoma, compiling the available literature data, and indicating the promising role of the NLR in this heterogeneous landscape of tumors. Specifically, an increased NLR in patients with cSCC is a proxy for the finding of advanced disease, indicating a potentially compromised course. The rather limited evidence presented herein, from four studies, is corroborated by a meta-analysis of seven retrospective studies that have addressed SSC in the epithelia of male external genitals [68]. Saputra et al., by employing the studies’ specific cut-off values for the NLR (ranging from 2.60 to 3.59) as a dichotomous predictor, revealed that a higher than cut-off NLR value is an independent predictor for the presence of lymph node metastases (OR = 6.67, 95%CI: 2.44–18.22; *p* < 0.01) and of shorter cancer-specific survival (HR = 2.15, 95%CI: 1.23–3.73; *p* < 0.01); however, not of the overall survival of the patient (HR = 1.69, 95%CI: 0.95–3.00, *p* = 0.07) [68]. Also, in a recent cohort study of 96 patients with penile SCC, a higher NLR was independently associated with shorter progression-free survival, while patients with an NLR > 3.0 were at higher risk of experiencing more advanced disease [69].

Similarly, in a retrospective study including 64 patients who underwent complete surgical staging for vulvar SCC, a preoperative NLR > 2.81 was more common in patients with, compared to those without, lymph nodal involvement (60.7% vs. 5.6%; RR = 10.9, 95%CI: 2.7–43.4; *p* < 0.001) [70]. Moreover, the mean tumor sizes were 4.2 ± 2.3 cm in the NLR > 2.81 group compared to 2.1 ± 1.2 cm in the NLR ≤ 2.81 group (*p* = 0.001) [70]. Finally, in a retrospective study of 21 patients with SCC of the auditory canal, most of them of the external auditory canal (EAC: *n* = 18; the rest *n* = 3 of middle ear), who received radiotherapy with or without surgery or systemic therapy, the OS differed significantly between patients with a high vs. low pretreatment NLR (*p* = 0.037), suggesting that the NLR has a significant prognostic impact on treatment outcomes in patients with locally advanced SCC of the EAC [71]. Notably, convincing data also support a prognosticator role for the NLR in SCC arising on diverse extracutaneous localizations, including the oropharynx and head and neck regions [72,73,74,75,76,77], esophagus [78,79,80], and anus [81,82]. The above evidence, taken together, indicates that the NLR seems to be a reliable biomarker of disease aggressiveness (poorer progression-free survival and higher cancer specific mortality) in patients with SCC, including patients with cSCC [83].

A meta-analysis of 23 studies that compiled the data of 4480 patients with STS reported that an increased NLR was significantly associated with decreased OS (HR = 2.01) and DFS (HR = 1.90), and increased disease-specific mortality (OR = 1.40). However, no data detailing the anatomic tissue of origin of these tumors were presented [84]. Nevertheless, at least some of the reviewed studies included cases of STSs of cutaneous origin, like dermatofibrosarcoma protuberans (DFSP). Que et al. [85], in their analysis of 222 patients with STSs of various histological types, discriminate between ‘superficial’ and ‘deep’ tumors, not specifying, however, how many of the former could have taken their origin from skin structures. Yet, they included cases of entities either typical for a cutaneous tumor (*n* = 28 cases of DFSP, 12.6% of the sample) or tumors that are relatively frequently seen in the skin (malignant fibrous histiocytoma, MFH). Interestingly, an elevated NLR was significantly associated with, among other parameters, deep tumor location and shorter DFS and OS. Regarding cutaneous sarcomas, in a subgroup analysis stratified according to histological type, the NLR was not a significant biomarker for the umbrella entity of fibrosarcomas, including DFSP; however, it was for the MFH/pleomorphic sarcoma cases. 

It is worth noting that, in contrast to the cutaneous solid tumors, the available data do not decisively support a biomarker role for the NLR in primary CTCL, at least not in the earlier MF disease stages. However, methodological differences between the studies, particularly the stage composition of the included patients, might at least partly account for the conflicting conclusions. Moreover, even though MF is a relatively indolent disease, studies that, in addition to presenting biomarker associations with disease stage and progression in stage, would also report on the correlation of biomarker levels and OS of the patients are important for the assessment of the utility of the NLR and similar hematological markers in approaches to patients with MF.

Immunotherapy, and particularly treatment with immune checkpoint inhibitors (ICIs), has demonstrated clinical benefits for a wide range of cancer types and also plays an increasingly important role in the management of patients with advanced cutaneous malignancies, including malignant melanoma, advanced keratinocyte skin cancer, and Merkel cell carcinoma [86,87,88,89]. However, the treatment outcomes are strongly patient-variable, making essential the search for factors that may predict individual responses to immunotherapy. Accumulating evidence clearly indicates that a higher baseline NLR has the features of an agnostic biomarker of the poor outcome of ICI treatment, i.e., across all cancers independent of their types [90,91]. In a retrospective cohort study, a higher NLR was significantly associated with poorer OS and progression-free survival (PFS), and lower rates of response and clinical benefit, after ICI therapy across multiple cancer types (16 different cancer types, *n* = 1714 patients). Furthermore, the combination of the NLR with tumor mutational burden (TMB) significantly improved the prediction of the probability of benefit from ICI (OR = 3.22; 95%CI: 2.26–4.58; *p* < 0.001 by comparing the NLR low/TMB high group to the NLR high/TMB low group) [92]. 

To date, regarding cutaneous malignancies beyond the malignant melanoma, there is only scattered explicit evidence that NLR is a predictor of ICI response for patients with cSCC treated with cemiplimab [42] and MCC treated with first-line prembulizumab [53]. However, for advanced KSC, concluding from data available for extracutaneous SCC, we would expect that the NLR will prove a prognostic factor for cSCC too. Thus, a meta-analysis that compiled the findings of 14 studies and included 929 patients with skin-adjacent, head and neck SCC (HNSCC) reported that a higher NLR was associated with poorer disease control (OR = 0.30, 95%CI: 0.12–0.74), PFS (HR = 2.15, 95%CI: 1.44–3.21), and OS (HR = 2.03, 95%CI: 1.50–2.74). The authors concluded that the NLR may adequately predict treatment outcomes in patients receiving ICI schedules for HNSCC [74].

This review focused on the systematic collection, analysis, and synthesis of the available evidence, regarding the hematological indexes, and particular the role of NLR as prognostic factors in cutaneous tumors other than melanoma. Among the strengths of this review is the rigorous methodology applied, limiting the risk of bias and, therefore, improving the quality of the evidence presented. In addition, the utilization of a tool to grade the confidence of the reported results further improved the conducted study analysis.

Nevertheless, this review is still subject to limitations. Of note is the relatively small number of available studies, especially for subgroup analysis according to the skin cancer type. The different molecular pathways, involved in the tumorigenesis of the skin cancers analyzed, also impact heterogeneity, although we aimed to analyze the role of the NLR separately for each skin cancer type.

Overall, further research is anticipated to shed light onto the evolving landscape of cutaneous oncology and the molecular mechanisms involved, enabling the integration of hematological biomarkers, such as the NLR, into clinical practice. Epidemiological data and better comprehension of these tumors’ biology are expected to improve the diagnostic accuracy and predictive precision of the biomarkers applied, towards an improved, more personalized approach to treatment, based on the principles of precision medicine.

## 5. Conclusions

This systematic review concludes that a higher NLR, a robust indicator of adverse prognosis across the common metastatic cancers including malignant melanoma, is a promising biomarker of tumor aggressiveness in all advanced-stage non-melanoma skin cancers (cSCC, EMPD, MCC, and certain cutaneous sarcomas). A distinct exception, with still inconclusive evidence is CTCL, although there are some data indicating that a higher NLR may predict poorer prognosis in patients with notably earlier stages of MF. Finally, given the promising data concerning the impact of the NLR as a prognosticator of the outcome of treatment with current immunotherapies, there is presently a need for studies focusing on the role of the NLR in the framework of the treatment of advanced skin cancer.

## Figures and Tables

**Table 2:** Summary of findings of hematologic biomarkers in cutaneous malignancies (excl. malignant melanoma). ↑ and ↓ indicate higher and lower index values, respectively. ND: no data available.

	Neutrophil-to-Lymphocyte Ratio (NLR)	Platelet-to-Lymphocyte Ratio (PLR)	Pan-Immune Inflammation Value (PIV)
cutaneous Squamous Cell Carcinoma (cSCC)	↑ NLR is associated with poor disease activity. ↑ NLR is associated with SLN positivity. ↑ NLR is associated with advanced stage cSCC. ↓ Baseline NLR is correlated with better response to Cemiplimab treatment. ↓ Baseline NLR correlated with non-advanced cSCC.	↓ Baseline PLR correlated with better response to Cemiplimab treatment.	ND
Extramammary Paget’s Disease (EMPD)	↑ NLR associated with metastatic EMPD. ↑ NLR associated with SLN positivity. NLR identified as independent prognostic factor for overall survival. NLR identified as predictor of lymph node metastasis. NLR identified as independent predictor of survival among EMPD patients with metastatic disease.	ND	ND
Merkel Cell Carcinoma (MCC)	↑ NLR is associated with increased disease-specific mortality. ↓ NLR is associated with better response rates to first-line pembrolizumab treatment. ↓ NLR is associated with longer patient survival.	ND	↑ PIV is observed in MCC patients.
Cutaneous Angiosarcoma	↑ NLR is associated with shorter overall survival.	↑ PLR is associated with patients with angiosarcoma of the face and scalp (ASFS).	ND
Mycosis fungoides (MF)	Contradicting results on the use of NLR as a biomarker on MF prognosis, staging, or progression.	ND	ND

## Data Availability

No new data were created or analyzed in this study. Data sharing is not applicable to this article.

## Supplementary Materials

The following supporting information can be downloaded at: https://www.mdpi.com/article/10.3390/cancers16051044/s1, Table S1. PRISMA 2020 checklist. Table S2. Keywords and MeSH terms used in different databases in the literature search. Table S3. Quality assessment of non-comparative studies. Table S4. Quality assessment of comparative studies. Table S5. Traffic light plot for the assessment of risk of bias for the comparative studies.
